# Utility of Digital Phenotyping Based on Wrist Wearables and Smartphones in Psychosis: Observational Study

**DOI:** 10.2196/56185

**Published:** 2025-02-05

**Authors:** Zixu Yang, Creighton Heaukulani, Amelia Sim, Thisum Buddhika, Nur Amirah Abdul Rashid, Xuancong Wang, Shushan Zheng, Yue Feng Quek, Sutapa Basu, Kok Wei Lee, Charmaine Tang, Swapna Verma, Robert J T Morris, Jimmy Lee

**Affiliations:** 1 North Region, Institute of Mental Health, Singapore, Singapore; 2 Ministry of Health Office for Healthcare Transformation, Singapore, Singapore; 3 Department of Psychosis, Institute of Mental Health, Singapore, Singapore; 4 Yong Loo Lin School of Medicine, National University of Singapore, Singapore, Singapore; 5 Lee Kong Chian School of Medicine, Nanyang Technological University, Singapore, Singapore

**Keywords:** schizophrenia, psychosis, digital phenotyping, wrist wearables, mobile phone

## Abstract

**Background:**

Digital phenotyping provides insights into an individual’s digital behaviors and has potential clinical utility.

**Objective:**

In this observational study, we explored digital biomarkers collected from wrist-wearable devices and smartphones and their associations with clinical symptoms and functioning in patients with schizophrenia.

**Methods:**

We recruited 100 outpatients with schizophrenia spectrum disorder, and we collected various digital data from commercially available wrist wearables and smartphones over a 6-month period. In this report, we analyzed the first week of digital data on heart rate, sleep, and physical activity from the wrist wearables and travel distance, sociability, touchscreen tapping speed, and screen time from the smartphones. We analyzed the relationships between these digital measures and patient baseline measurements of clinical symptoms assessed with the Positive and Negative Syndrome Scale, Brief Negative Symptoms Scale, and Calgary Depression Scale for Schizophrenia, as well as functioning as assessed with the Social and Occupational Functioning Assessment Scale. Linear regression was performed for each digital and clinical measure independently, with the digital measures being treated as predictors.

**Results:**

Digital data were successfully collected from both the wearables and smartphones throughout the study, with 91% of the total possible data successfully collected from the wearables and 82% from the smartphones during the first week of the trial—the period under analysis in this report. Among the clinical outcomes, negative symptoms were associated with the greatest number of digital measures (10 of the 12 studied here), followed by overall measures of psychopathology symptoms, functioning, and positive symptoms, which were each associated with at least 3 digital measures. Cognition and cognitive/disorganization symptoms were each associated with 1 or 2 digital measures.

**Conclusions:**

We found significant associations between nearly all digital measures and a wide range of symptoms and functioning in a community sample of individuals with schizophrenia. These findings provide insights into the digital behaviors of individuals with schizophrenia and highlight the potential of using commercially available wrist wearables and smartphones for passive monitoring in schizophrenia.

## Introduction

Schizophrenia is a debilitating condition affecting at least 20 million individuals worldwide [1]. This lifelong condition [2] is frequently associated with impairments in personal, social, occupational, and other important areas of daily function [3]. Despite advancements in pharmacology and psychosocial interventions, recovery rates among patients have not improved [4], and the high rate of relapse places a heavy burden on patients and their caregivers [5].

In present-day psychiatric care, the clinical status of patients is determined at periodic consultations via clinician interviews and self-reported questionnaires. The episodic nature of these visits and the removal of patients from their natural environment result in limited assessments that do not always reflect the state of patients in different environments and over varying periods of time [6]. Moreover, there is a risk of unreliable self-reporting in patients with schizophrenia, influenced by cognitive impairments, biases, and self-reporting confounders [7].

Digital phenotyping may allow care teams to circumvent these limitations. Digital phenotyping is defined as the moment-by-moment quantification of the individual-level human phenotype in situ using data from smartphones and other personal digital devices [8,9]. Through personal digital devices, insightful and objective longitudinal data, such as on sleep and mobility, can be unobtrusively and continuously collected from patients [10].

There has been growing interest in digital phenotyping in psychiatry [11,12]. People with schizophrenia have shown willingness to use smartphones to monitor their condition [13]. Furthermore, preliminary trials have shown that the long-term use of wearable technology is feasible and acceptable by people with schizophrenia and that paranoia toward devices was not a significant barrier [14-16].

There is also some evidence that wearable and mobile devices can deliver clinically relevant information in real-world contexts. Movement data estimated from a 3-axis accelerometer were negatively correlated with negative symptoms assessed with the Positive and Negative Syndrome Scale (PANSS) in patients with schizophrenia [17]. The number of steps was negatively correlated with PANSS positive-factor, negative-factor, general subscales, and total score, in patients with schizophrenia in an inpatient setting [18]. Sleep duration estimated from mobile phone–based accelerometer data was moderately correlated with subject self-assessment of sleep duration [19]. Geolocation and motor activity inferred from gyroscopes and data from the ambient light sensor, accelerometer, and other sensors in smartphones were used as indices of negative symptoms [20,21]. Data from anonymized call and text message logs may be potential markers for social functioning, and data on screen touches, such as typing speed, may provide novel clinical data about a user’s cognitive functioning [9].

Despite this evidence, past studies have noteworthy limitations, such as small sample sizes [17,22], nonclinical samples [23,24], being conducted in closed-ward settings [18], and having used made-for-research wearable devices [22], which would affect the generalizability of the research findings or scaling up to larger populations. To date, few studies have reported digital phenotyping measures collected with both low-cost, commercially available digital wearable devices and smartphones among patients with schizophrenia in a community setting.

The aim of this study was to provide an initial examination of the associations between multimodal digital markers and clinical outcomes in patients with schizophrenia.

## Methods

### Setting and Participants

The Health Outcomes via Positive Engagement in Schizophrenia (HOPE-S) study is a prospective observational study conducted at the Institute of Mental Health, Singapore, with recently discharged individuals with schizophrenia spectrum disorders. Details of the study have been previously reported [25]. In brief, participants were followed up for up to 6 months.Clinical, cognitive, and functioning data were collected at intervals of every 6 weeks, and digital data from smartphones and wrist wearables were collected continuously over the follow-up period. The clinical, cognitive, and functioning data collected at baseline and the digital data collected in the first week after study enrollment were used for this study. The study aimed to examine the associations between clinical, cognitive, functioning, and digital data to develop prediction algorithms that might detect clinically significant outcomes. We recruited 100 English-speaking patients aged 21‐65 years between November 2019 and January 2023. One participant requested to be withdrawn from the study after completing the baseline assessment due to objections from family members. Of the remaining participants (n=99), 63 were diagnosed with schizophrenia, 12 with brief psychotic disorder, 11 with schizophreniform disorder, 5 with schizoaffective disorder, 5 with delusional disorder, and 3 with other specified psychotic disorder.

### Ethical Considerations

Ethics approval was obtained from the National Healthcare Group’s Domain Specific Review Board, Singapore (2019/00720). Written informed consent was obtained from all participants after the study was fully explained. Data were anonymized and encrypted to protect patient privacy. Participants were reimbursed SGD 50 (US $37.19) on completion of the baseline visit for their time, inconvenience, and transportation costs.

### Clinical Symptom Severity Measures and Scales

Assessment data from baseline were used for the present analyses. Symptom severity was assessed on the PANSS [26]. The PANSS is a 30-item clinical scale measuring positive symptoms, negative symptoms, and general psychopathology. PANSS factor scores, including positive (PANSS_Pos), negative (PANSS_Neg), cognitive/disorganization (PANSS_Cog/Dis), depression/anxiety (PANSS_Dep/Anx), and hostility (PANSS_Hos), were calculated in accordance with a previously validated factor structure [27].

Negative symptoms were measured with the Brief Negative Symptom Scale (BNSS) [28], a well-established 13-item instrument designed to measure 5 negative symptom domains, including asociality, anhedonia, avolition, blunted affect, and alogia [29].

Depressive symptoms were assessed on the Calgary Depression Scale for Schizophrenia (CDSS) [30], a 9-item scale widely used for assessing depression in schizophrenia that differentiates between depression symptoms and the negative and positive symptoms in schizophrenia.

Neurocognition was assessed with the Brief Assessment of Cognition in Schizophrenia (BACS) [31], a cognitive battery that assesses attention, verbal and working memory, motor and processing speed, verbal fluency, and executive function. A composite *z* score was obtained and used for analysis [31]. The Social and Occupational Functioning Assessment Scale (SOFAS) [32] was used to evaluate the patients’ social and occupational functioning.

### Digital Data Collection and Preprocessing

Digital data were collected from both wrist wearables and smartphones. Each participant was issued a Fitbit Charge 3 or 4 (Fitbit Inc) at the beginning of the study. Participants were required to wear the Fitbit every day during the study period, including when asleep. The Health Outcomes through Positive Engagement and Self-Empowerment (HOPES) app [33] was installed on a participant’s smartphone if the device used the Android operating system. In the circumstance that a participant’s phone was not compatible with the HOPES app, we provided a phone for use during the study period. In this case, study coordinators aided in the patient’s migration to the study phone, and the patient was instructed to use only the study phone as their primary mobile device during the study. Data collected from the wrist wearables and smartphones were automatically synchronized with the HOPES app and subsequently sent to the research premises for storage and further analysis. Data collected by the Fitbit included data from the heart rate monitor, pedometer (step counts), and sleep sensor, including sleep stages. Data collected from the smartphone included locational mobility features (through GPS), touchscreen tapping speed, and sociability indices from texts and calls (via both the mobile service provider and WhatsApp). Significant steps were taken to ensure data privacy, validate data quality, and engineer better features, which are covered in detail in a publication by Wang et al [33]. The subset of data analyzed in this paper is described in Multimedia Appendix 1. Table 1 summarizes the resulting digital measures used in our analyses.

**Table 1.** Digital measures collected by Fitbit wearables and smartphones.

Daily digital measures	Data source	Description
*total_sleep_hrs*	Wearable	Total hours of sleep, including naps but not including the “wake” stage (see below).
*sleep_hrs_wake*	Wearable	Total hours of sleep classified as the “wake” sleep stage by the Fitbit, estimated to be the time in bed not sleeping (ie, falling asleep, after waking up, and awaking during the night).
*sleep_hrs_light*	Wearable	Total hours of sleep classified as the “light” sleep stage by the Fitbit.
*sleep_hrs_deep*	Wearable	Total hours of sleep classified as the “deep” sleep stage by the Fitbit.
*sleep_hrs_REM*	Wearable	Total hours of sleep classified as the “REM” (rapid eye movement) sleep stage by the Fitbit.
*sleep efficiency*	Wearable	Sleep efficiency score from the Fitbit for the main sleep segment.
*HR_asleep*	Wearable	Average of heart rate samples while asleep, as measured by the Fitbit heart and sleep monitors.
*total steps*	Wearable	Total steps taken, as measured by the Fitbit.
*dist_travelled*	Smartphone	Total distance traveled, as computed by GPS processing routines.
*mode_intertap_dist*	Smartphone	Mode of a rolling 7-day distribution of intertap durations, excluding taps in games (a higher value indicates a slower tapping speed).
*total_msg_sent*	Smartphone	Total messages (texts and images) sent via mobile service provider and WhatsApp.
*screen_time*	Smartphone	Total time of smartphone use as represented by the power state logs.

For each daily digital measure, data collected during the 7 days following the baseline visit (not including the data on the day of the baseline visit) were used. The 1-week duration should account for any day-of-week effects in the digital measures. Digital markers were taken as the average of the observations for each digital measure over the week of interest, except for the *mode_intertap_dist* measure, for which the last valid (ie, nonmissing) value was taken since this is already a 7-day rolling window statistic. We required at least 4 valid observations in the week-long observation window to produce a nonnull marker.

Successful data collection rates for the wearables as the fraction of hour-long windows with at least 1 successfully collected heart rate sample in the window of study (ie, the 7 days [168 hours] following the baseline visit) were calculated. A successful data collection rate for the smartphones was calculated similarly using the ambient light sensor. Note that these 2 data sources were continually sampled by each device regardless of user action, so long as the participant was compliant, that is, the wearable was correctly worn, and the phone was powered on with the app running in the background.

### Statistical Analysis

Descriptive statistics on clinical measures, digital data measures, and successful data collection rates were analyzed. The successful data collection rates were compared between groups of participants who used study phones and their own phones with the Mann-Whitney *U* test. As an exploratory analysis to discover possible digital markers as factors associated with different clinical measures, a multiple linear regression was performed independently for each pair of digital markers (independent variable) and clinical measure (dependent variable). In each regression model, age was included as a covariate along with an interaction term with the digital measure, which has been reported as a significant factor in relapse behavior, psychosocial functioning, and psychotic symptoms [34,35]. Because a significant difference in digital data collection rates was noticed between participants using a study-provided phone and those using their own phone, we also included a variable indicating whether the participant was using a study-provided phone (along with its interaction with the digital measure) in all models regressing onto a digital measure from the smartphone. To easily compare the relative effect sizes between different measures, both the digital markers and clinical scales were standardized across the population; an intercept term was therefore not included in the models. Due to the particularly skewed distributions of the *distance_travelled* and *total_msg_sent* features, a log(1+x) transform was applied to these features before standardization. As this was an exploratory analysis attempting to uncover trends in the digital markers, multiple testing corrections were not used. A heat map was used to show effect sizes and visualize trends across digital markers and clinical scales.

## Results

### Sample Characteristics

The demographic and clinical characteristics of the study sample are summarized in Table 2.

**Table 2.** Demographic and clinical characteristics of the study sample (n=99).

Characteristics	Values
**Sex, n (%)**	
Female	57 (57.6)
Male	42 (42.4)
**Marital status, n (%)**	
Single	85 (85.9)
Married	7 (7.1)
Separated or divorced	7 (7.1)
**Ethnicity, n (%)**	
Chinese	73 (73.7)
Malay	17 (17.2)
Indian	6 (6.1)
Other	3 (3.0)
**Currently on antipsychotics, n (%)**	
Yes	97 (98.0)
No	2 (2.0)
Age (years), mean (SD)	29.88 (6.72)
Years of education, mean (SD)	14.07 (2.27)
Duration of illness (years), mean (SD)	2.61 (4.00)
Antipsychotic dose^a^ (mg), mean (SD)	484.37 (375.55)
**PANSS** ^**b**^ ** score, mean (SD)**	
Total	51.11 (9.97)
Positive	8.49 (3.82)
Negative	13.20 (4.08)
Cognitive/disorganization	10.55 (2.50)
Depression/anxiety	8.85 (3.43)
Hostility	5.40 (1.89)
**BNSS**^**c**^ **score, mean (SD)**	
Total	20.90 (9.30)
Anhedonia	5.51 (2.76)
Asociality	3.34 (1.55)
Avolition	3.30 (1.89)
Blunted	5.77 (3.89)
Alogia	2.82 (2.61)
CDSS^d^ total score, mean (SD)	12.31 (3.55)
SOFAS^e^ score, mean (SD)	53.96 (11.85)
BACS^f^ score, mean (SD)	−1.31 (1.36)

a Antipsychotic dose was computed as total daily chlorpromazine equivalent (n=91).

b PANSS: Positive and Negative Syndrome Scale.

c BNSS: Brief Negative Symptom Scale.

d CDSS: Calgary Depression Scale for Schizophrenia.

e SOFAS: Social and Occupational Functioning Assessment Scale.

f BACS: Brief Assessment of Cognition in Schizophrenia.

### Digital Data Collection Rate and Digital Data Profile

In total, 41 and 58 participants were issued a Fitbit Charge 3 and Charge 4, respectively. Thirty-nine participants used a smartphone provided by the study at the baseline visit, while the rest used their own phone. The successful data collection rate in the week following the baseline visit was 91% (SD 22%) for the Fitbit and 82% (SD 32%) for the smartphone. Those using a study-provided phone had a significantly higher successful data completion rate than those using their own phone (mean 91% vs 77%; Mann-Whitney *U* test: *P*<.001). Sensor-specific successful data collection rates are also provided in Multimedia Appendix 2. The sample size of valid digital measures (n=99), along with the means and SDs, are presented in Table 3.

**Table 3.** Summary of digital markers.

Visit-level digital markers	Values, mean (SD)
*Total_sleep_hrs* (n=92)	8.76 (2.13)
*Sleep hrs-wake* (n=91)	0.83 (0.30)
*Sleep hrs-light* (n=91)	5.32 (1.71)
*Sleep hrs-REM* (n=91)	1.68 (0.51)
*Sleep hrs-deep* (n=91)	1.23 (0.39)
*Sleep_efficiency* (n=91)	0.93 (0.03)
*Total_steps* (n=96)	9186.91 (5413.21)
*Distance_travelled* (km) (n=87)	27.64 (34.59)
*HR_asleep* (bpm) (n=74)	69.94 (10.06)
*Mode_intertap_dist* (ms) (n=96)	596.06 (389.08)
*Total_msg_sent* (n=92)	38.02 (65.16)
*Screen_time* (hrs) (n=92)	4.74 (3.64)

As expected, several digital measures were correlated with one another (such as sleep stage measures). The correlation coefficients between digital measures are provided in Multimedia Appendix 3.

### Association Between Clinical Scales and Digital Markers

**Figure 1.**
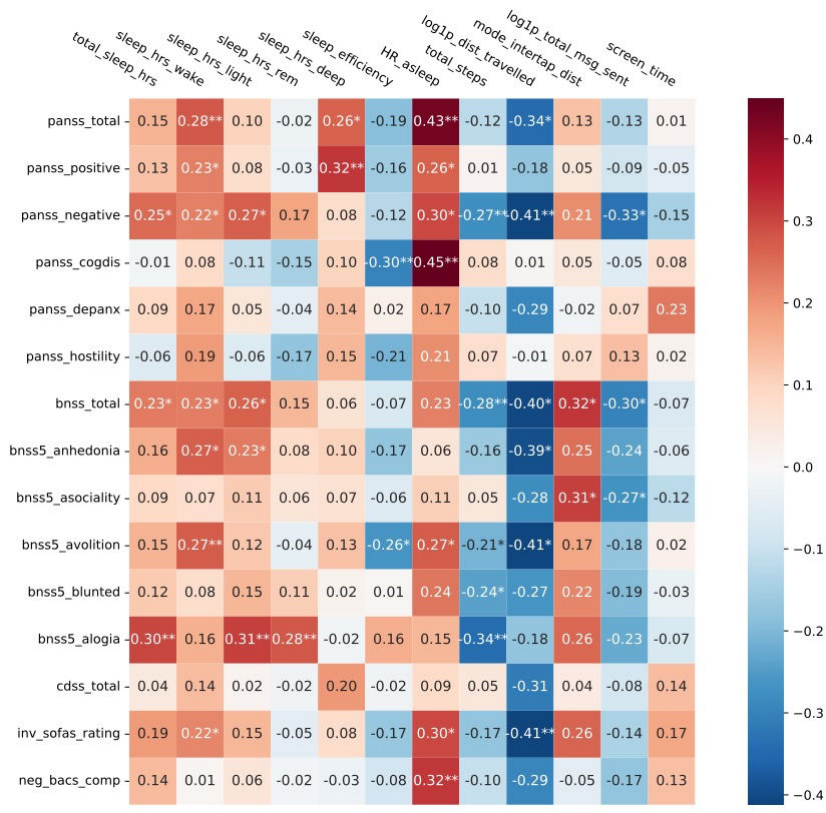
Regression coefficients between clinical scales and digital markers. Notes: A log(1+x) transformation was applied to *distance_travelled* and *total_msg_sent*. All clinical scales and digital markers were standardized prior to the regression. The Social and Occupational Functioning Assessment Scale (SOFAS) score was inverted (denoted as *inv_soafs_rating*, calculated as 100 minus the SOFAS rating) in the analyses to maintain a consistent direction among the scales and the digital markers, which aids visualization and interpretation. Analogously, the Brief Assessment of Cognition in Schizophrenia composite score was inverted (denoted as *neg_bacs_comp*) by taking the negative of its value. Ordinary least squares: *P_OLS_<.05, **P_OLS_<.01.

The linear regression coefficients of the digital measures are visualized for the different clinical measures as a heat map (Figure 1) at the mean age level (29.88 years) and for participants using their own phone. The numbers in the cells are the value of the regression coefficient, which also determines the cell color and intensity.

Starting with the symptoms and their associated digital biomarkers, first, we found that negative symptoms as assessed by both the BNSS and PANSS-negative were associated with the most digital measures (8 of the 12 being studied). Experiential deficits, that is, anhedonia and avolition, tended to be associated with less distance traveled and more hours spent awake in bed; fewer messages sent, a digital marker of sociability, tended to be associated with asociality. Expressive deficits, that is, affect blunting and alogia, tended to be negatively associated with steps taken. Alogia was additionally associated with sleep features such as total sleep hours and hours in light and rapid eye movement (REM) sleep. Second, PANSS-positive and SOFAS scores were associated with at least 3 different digital measures. Third, cognition and cognitive/disorganization symptoms were associated with 1 or 2 digital measures.

Among the digital measures, heart rate measured when asleep was associated with the most clinical measures. Specifically, a higher heart rate was associated with greater severity scores on PANSS-total and its factors (ie, positive, negative, and cognitive/disorganization) and BNSS avolition and asociality, as well as poorer functioning and cognitive performance. Time awake in bed and distance traveled were associated with mostly similar clinical measures, that is, negative symptoms, in particular anhedonia, avolition, and functioning. The only digital measure not significantly associated with a clinical measure was screen time.

## Discussion

### Principal Findings

In this study, we examined the associations between digital markers (collected via commercially available wrist-wearable devices and smartphones) and a comprehensive range of clinical assessments in a sample of 99 recently discharged patients with schizophrenia spectrum disorders. Notable patterns of associations for digital measures were observed with psychiatric symptoms, specifically negative symptoms, functioning, and cognitive performance.

Longer total sleep time was positively associated with negative symptom domains; sleep efficiency was negatively associated with BNSS avolition and the PANSS cognitive/disorganization score. These results are in line with previous studies that reported associations between negative symptom severity and sleep disturbances (such as hypersomnia and sleep satisfaction) collected through self-reporting [36,37] and as measured by actigraphy [22,38]. One study also found no correlation between sleep markers collected via actigraphy and negative symptoms assessed via the PANSS-negative symptom subscale [39]. In addition, whether and how each sleep stage is associated with symptoms in schizophrenia remains inconclusive. A previous study reported that REM sleep duration was correlated with the Brief Psychiatric Rating Scale (BPRS) total score but not with the constituent negative symptom score [40]; in contrast, this study found no association between REM sleep duration and PANSS-total score but found a positive association with alogia. Use of nonspecific negative symptom assessment scales such as PANSS and BPRS, variations in sleep instruments, and other clinical factors, such as duration of illness and medication status [41], likely contributed to the mixed results in the literature.

Several types of passive digital phenotyping measures are associated with negative symptoms, such as phone use data (call/text logs, screen time), GPS data (distance traveled), sleep data (total sleep duration), and physical activity (step count). These findings support the use of passively collected digital phenotyping data as objective measures of negative symptoms and functional outcomes. These measures also provide longitudinal data that are usually not available directly to clinicians. Our study found a negative association between physical activity (as measured by step count) and negative symptom severity, particularly BNSS avolition, blunted affect, and alogia. This is in line with previous research that showed associations between decreased activity or sedentary behavior and negative symptoms in schizophrenia [17,18,22,42-44].

Distance traveled, messages sent and touchscreen tapping speed yielded the strongest signals associated with negative symptoms, specifically experiential deficits such as avolition, anhedonia and asociality. These results are consistent with previous studies that reported greater GPS mobility was associated with lower negative symptom severity and better community functioning [20,45]. Social behaviors (such as duration of outgoing calls and number of text messages) have been found to be predictive of psychotic symptoms and relapse in schizophrenia [46,47]. Typing-related data have been reported to be associated with mood symptoms and cognition in people without psychosis [48-50], but no such association was found in our study. The current study appears to be the first to report significant associations between negative symptoms, specifically asociality, and a messaging log feature (ie, the number of messages sent out) as well as touchscreen tapping speed.

We found that increased heart rate while asleep was associated with increases in positive psychopathology and worse cognitive performance. The relationship between increased heart rate and a greater severity of positive symptoms has often been documented [51-53], but only one previous study reported this relationship for heart rate during sleep, supporting our findings [54]. These associations likely indicate altered autonomic functioning, as indexed by increased heart rate, in response to stressors such as manifestations of psychotic symptoms [51,55]. Similarly, altered autonomic functioning may affect cognition. While no studies have reported the association of increased heart rate during sleep with worsening cognition, reduced heart rate variability has been linked to worse cognitive scores in schizophrenia [56].

The successful data collection rates in this study were high—about 91% for the Fitbit devices and 82% for the smartphones, which is comparable to the compliance rates in previous studies [15,19]. The good data collection rates support the potential of wrist wearables and smartphones as longitudinal passive monitoring devices in people with schizophrenia. It should be noted that the enrolled sample was relatively young, with a mean age of 30 years, and this group of people may be more accepting of digital devices. The digital data collection rate from the smartphones was impacted by both the compliance of participants and technical factors, such as proper device functioning and syncing with the mobile app. Some smartphone models automatically suppress all app activity during the night to save battery power, preventing complete data collection from the phone. In addition, some participants were provided with a phone for the study because their personal phone was incompatible with the HOPES app, and it was often the case that the participant’s phone was too old. The above 2 points could explain why the data collection rate was higher with study-provided phones, as these (newer) phones could have exhibited better functioning.

Although there is a considerable amount of literature reporting associations between digital markers and clinical symptoms in populations of outpatients with schizophrenia, the majority collected digital data via only smartphones or actigraphy devices. Despite the expanding interest in using commercially available wearables in health care [57], studies using these devices in the medical field are still few. This study contributes such an investigation with a large clinical sample. Notably, the data reported in this study were not affected by Singapore’s COVID-19 movement control measures: none of the 1-week digital data collection periods under study overlapped with the movement control period.

### Limitations

There are some limitations to this study to be considered. The participants were relatively young (mean age: 30 years) and mildly ill (PANSS-total score: 51) [58] with a short duration of illness (2.6 years), which limits the generalizability of the results. Associations in older, chronic, and more severely ill patients might differ and should be further investigated. Due to the nature of this cross-sectional study, no causal relationships could be inferred. In addition, all participants included in this study were taking psychotropic medications with varying types and dosages, which may have had differential effects on cardiac function [59] and sleep [60], warranting further examination. Participants received financial incentives at timed intervals in the study, which might have positively affected adherence and data acquisition. Similar studies, however, noted that the majority of participants expressed a desire to continue using the wrist wearable without incentive after the study [19]. The longer-term use of wearable devices by individuals with schizophrenia should be further studied. Finally, because the regression analyses were exploratory in nature, and we aimed to detect all potential indicators of symptoms and functioning that could be useful in future studies, corrections for multiple testing were not applied before reporting significant associations.

Despite the limitations of the study, the patterns highlighted in the findings may guide future directions for research. The reported associations suggest that unobtrusively and passively collected digital phenotyping markers hold promise for continuous monitoring of clinical symptoms in people with schizophrenia between clinical visits. Digital phenotyping–enhanced care can improve the identification of patients in need of attention and may allow health care providers to delivery timely interventions [61].

### Conclusion

Our study found notable patterns of associations between a range of digital biomarkers and clinically important outcome measures in a community sample of individuals with schizophrenia spectrum disorders. These results support the use of commercial wrist wearables and smartphones in continuous but passive monitoring of the health status of individuals with schizophrenia. Future studies should explore longer-term digital phenotyping observations in naturalistic settings and their utility in predicting clinical events.

## Supplementary material

10.2196/56185 Multimedia Appendix 1 Data collection and feature processing in the Health Outcomes through Positive Engagement and Self-Empowerment (HOPES) platform.

10.2196/56185 Multimedia Appendix 2 Sensor-specific successful data collection rates.

10.2196/56185 Multimedia Appendix 3 Correlations between digital measures.

## Abbreviations

BACS: Brief Assessment of Cognition in Schizophrenia
BNSS: Brief Negative Symptom Scale
BPRS: Brief Psychiatric Rating Scale
CDSS: Calgary Depression Scale for Schizophrenia
HOPE-S: Health Outcomes via Positive Engagement in Schizophrenia
HOPES: Health Outcomes through Positive Engagement and Self-Empowerment
PANSS: Positive and Negative Syndrome Scale
REM: rapid eye movement
SOFAS: Social and Occupational Functioning Assessment Scale
