# Supplementary material for: Utility of Digital Phenotyping Based on Wrist Wearables and Smartphones in Psychosis: Observational Study
Source: JMIR Mhealth Uhealth. 2025 Feb 5;13:e56185. doi: 10.2196/56185 (PMC11822399; doi:10.2196/56185)
Supplement: Multimedia Appendix 1 [file mhealth-v13-e56185-s001.docx]

# Multimedia Appendix 1

**Data collection and feature processing in the HOPES platform**

In the HOPES platform [1], data collected from the wrist wearable is automatically synchronized with the installed HOPES smartphone App and subsequently sent, together with the data collected from the smartphone sensors to the cloud and research premises for storage and further analysis. All data collected and transferred from the Fitbit app and smartphone were de-identified using a separately generated trial subject identifier, with no personal identifiable information collected. The HOPES platform, data collection methodology, security/privacy preservation strategies, and feature engineering methods are all described in detail in [1]. Here we extract the details for the digital measures analysed in this article.

Heart

For heart rate, the raw data collected was a time series in beats-per-minute sampled every five seconds (at least) while the Fitbit is worn. If the Fitbit is not being worn, no heart rate data is sampled. We computed the daily mean of these heart rate samples while the patient was asleep (denoted as *HR_asleep*).

Sleep

Fitbit reports raw data as *sleep events*, a sequence of which starts and finishes when the wearer gets into and out of bed, respectively, and which were estimated by Fitbit. The start and end of each sleep event in the sequence was determined when the *sleep level* changes between being awake, in light sleep, in deep sleep, and in REM sleep. One of these sequences is identified by Fitbit as the *main sleep* sequence (which is differentiated from naps, for example). Short sleep periods of less than three hours during the day are not collected by Fitbit. Fitbit’s data API does not define a day-boundary or assign sleep segments to a particular day, and so this day-boundary must be specified for analyses at the daily data level. We used 15:15 as the boundary point between days, to avoid splitting the main sleep segment; in particular, 15:15 is the midway point between the average main sleep start time, 23:15, and the average main sleep end time, 7:15, observed in Singapore [2]. Some days have no sleep events recorded, which is ambiguous because the participant could either have not slept (which may be a common and indicative event amongst this population) or did not wear their Fitbit to sleep. We therefore declare sleep data to be *missing* on days in which heart rate data (which is sampled roughly every 5 seconds while the Fitbit is worn) is missing for more than three hours (at any point during the day), as this could be a period the participant slept but did not wear the Fitbit. If heart rate data is not missing, then we declare the total amount of sleep on that day to be zero hours. The choice of three hours as a threshold matches Fitbit’s own threshold for recording sleep events, mentioned earlier.

Steps

For steps, the raw data collected was a time series of the number of steps per minute (sampled every minute). We summed the samples over a day to get the total number of steps. We note, however, that Fitbit will record samples of zero steps even if the Fitbit is not worn. We therefore follow a similar strategy as with sleep, where we compare the sampled step count window against the heart rate data. If heart rate data is missing during the same window, then we drop the sample.

GPS

Raw GPS coordinates are collected by the HOPES App (a feature inherited from Beiwe), which are then obfuscated by random, participant-specific offsets, and only aggregated/summarizing features are processed, such as the radius of gyration analysed in this paper.

Messaging

The HOPES App can record the number of messages (both text and images) sent but does not record content. The number of messages sent via both the mobile service provider and WhatsApp are summed together.

Tapping speed

The HOPES App records taps on the touchscreen and their timestamps, from which we compute inter-tap durations (i.e., the length of time between taps). We accumulate these inter-tap durations over a seven-day window (by filtering out durations longer than 3s because those typically do not reflect the speed of typing text, rather they reflect the speed of navigation and thinking which depends on the content which the app does not capture) and find the peak of the power law distribution. We found that a seven-day rolling window was required to populate the inter-tap duration distributions with enough data to form a robust statistic. These inter-tap duration distributions are commonly used to characterize tapping speed and error rate which have been shown to be indicative of mood, behaviour, and cognitive functions [3, 4].

Screen time

The HOPES App records the power state logs of screen on and off events with timestamps. Using these records, we can compute how long the screen was turned on (presumably being used by the participant).

**References**

1. Wang X, Vouk N, Heaukulani C, Buddhika T, Martanto W, Lee J, Morris R. HOPES: An Integrative Digital Phenotyping Platform for Data Collection, Monitoring, and Machine Learning. J Med Internet Res. 2021; 23(3): e23984. DOI: 10.2196/23984
2. Walch OJ, Cochran A, & Forger DB. (2016). A global quantification of “normal” sleep schedules using smartphone data. Sci Adv. 2016; 2(5): e1501705. DOI:[10.1126/sciadv.1501705](https://doi.org/10.1126/sciadv.1501705)
3. Ross MK, Demos AP, Zulueta J, Piscitello A, Langenecker SA, McInnis M, et al. Naturalistic smartphone keyboard typing reflects processing speed and executive function. Brain Behav. 2021 Nov; 11(11): e2363. DOI: 10.1002/brb3.2363. PMID: 34612605; PMCID: PMC8613429.
4. Stange JP, Zulueta J, Langenecker SA, Ryan KA, Piscitello A, Duffecy J, et al. Let your fingers do the talking: Passive typing instability predicts future mood outcomes. Bipolar Disord. 2018 May; 20(3): 285-288. Doi: 10.1111/bdi.12637. PMID: 29516666. PMCID: PMC5940490.
