# Supplementary material for: Utility of Digital Phenotyping Based on Wrist Wearables and Smartphones in Psychosis: Observational Study
Source: JMIR Mhealth Uhealth. 2025 Feb 5;13:e56185. doi: 10.2196/56185 (PMC11822399; doi:10.2196/56185)
Supplement: Multimedia Appendix 2 [file mhealth-v13-e56185-s002.docx]

Multimedia Appendix 2

**Sensor-specific successful data collection rates**

We compute a data collection rate for each of the sensors as follows. For the accelerometer, ambient light sensor, and heart rate monitors, we compute the fraction of hour-long windows with at least one reading successfully collected in the window of study (i.e., the seven days following the baseline visit, which consists of 168 hours). For all other features, we check whether there is at least one reading every day. Take note that we often cannot distinguish whether missing data during particular periods are due to participants failing to comply with the study requirements (i.e., not wearing the wrist device, somehow preventing synchronization of the wrist device with the App regularly, or stopping/closing the App in the background, etc.) or to the Android operating system’s characteristics (e.g., suppressing App wake-ups in the night to save power). We therefore merely call this a *data collection rate*. What’s more, we note that the SMS log, Call log, WhatsApp message log, and WhatsApp call log all record no entries if no texts or calls occurred (as appropriate), which is the same outcome if a participant isn’t study compliant. We therefore cannot distinguish these two events.

Table S1 Successful data collection rate by sensor

| **Data completion rate in week following baseline visit** | **mean (SD)** |
| --- | --- |
| Accelerometer | 0.57 (0.26) |
| Ambient light sensor | 0.82 (0.32) |
| GPS | 0.89 (0.26) |
| Power state | 0.93 (0.22) |
| SMS log | 0.60 (0.31) |
| WhatsApp msg log | 0.81 (0.32) |
| Taps log | 0.91 (0.25) |
| Accessibility log | 0.90 (0.25) |
| Call log | 0.55 (0.33) |
| Heart | 0.91 (0.22) |
| Sleep | 0.90 (0.24) |
| WhatsApp call log | 0.26 (0.30) |
| Pedometer | 0.98 (0.13) |
