# Supplementary material for: Utility of Digital Phenotyping Based on Wrist Wearables and Smartphones in Psychosis: Observational Study
Source: JMIR Mhealth Uhealth. 2025 Feb 5;13:e56185. doi: 10.2196/56185 (PMC11822399; doi:10.2196/56185)
Supplement: Multimedia Appendix 3 [file mhealth-v13-e56185-s003.docx]

# Multimedia Appendix 3

**Correlations between digital measures**

In Figure S1, we display a heatmap of the Pearson correlation coefficients between the (possibly power-transformed) digital measures. In particular, a Yeo-Johnson power transformation (implemented through the scikit-learn Python package) is applied if a Shapiro-Wilks test produces a p-value less than 0.5 (indicating that the measure is not well-modelled by a Gaussian distribution). Cases with missing data are dropped.

| Figure S1. Pearson correlation coefficients between digital measures. |
| --- |
|  |
| Notes:   - The color of the cell represents the value of the Pearson correlation coefficient (also printed in the cell) between the possibly power-transformed variables. - A *log(1+x) transformation was applied to Distance_travelled and Total_msg_sent.* - Correlations with $p_{\mathrm{pearson}}<0.05$ and $p_{\mathrm{pearson}}<0.01$ are highlighted with a * and **, respectively, where $p_{\mathrm{pearson}}$ is the usual p-value computed for Pearson correlation coefficients. |
